# Fabrication of Woven Jute Fiber Epoxy Bio-Composites through the Epoxy/Thiol-Ene Photopolymerization Technique

**DOI:** 10.3390/polym15010060

**Published:** 2022-12-23

**Authors:** Ricardo Acosta Ortiz, Roberto Yañez Macías, José de Jesús Ku Herrera, Aida Esmeralda García Valdez

**Affiliations:** Centro de Investigación en Química Aplicada, Blvd Enrique Reyna # 140, Saltillo 25294, Coahuila, Mexico

**Keywords:** bio-composites, photopolymerization, epoxy/thiol-ene, woven jute fibers, silane functionalization

## Abstract

An eco-friendly epoxy/thiol-ene photopolymerization (ETEP) process was employed to prepare epoxy bio-composites using a commercial biobased epoxy resin and a woven jute fabric as reinforcement. In this process the components of the thiol-ene system, an allyl-functionalized ditertiary amine curing agent, a multifunctional thiol and a radical photoinitiator, were added to the epoxy resin to produce a polyether–polythioether crosslinked co-network. Moreover, the jute fibers were functionalized with thiol groups using the 3-mercaptopropyl (trimethoxysilane) with the purpose of creating a chemically bonded polymeric matrix/fiber system. The obtained bio-composites prepared with the thiol-functionalized cellulose fibers exhibited an increase up to 52% and 40% in flexural modulus and strength with respect to the non-functionalized counterparts. Under the three-point bending loadings, the composites displayed higher deformation at break and toughness due to the presence of polythioethers in the co-network. The prepared bio-composites developed in this work are excellent candidates to extend the use of cellulose fibers for structural applications.

## 1. Introduction

The search for novel, environmentally friendly and sustainable high-performance materials has encouraged the research on epoxy bio-composites, employing different types of cellulose fibers [1,2]. The use of natural plant-fibers is very attractive due to their low cost, high strength, low density, high specific strength and modulus, biodegradability and good thermal and acoustical insulation [3,4,5]. Additionally, the mechanical properties of some plant-fibers can even compete with those of the glass-fibers [6,7]. Different types of plant-fibers have been used as reinforcement to prepare epoxy bio-composites such as flax [8,9], sisal [10,11], jute [12,13], bamboo [14,15], sugar cane bagasse fiber [16], banana fiber [17,18] and kenaf fiber [19], to name some examples.

The epoxy bio-composites have found multiple applications in different areas such as in the aerospace, nautical, automotive, packaging and building industries [20]. For instance, in the automotive industry, bio-composites have become increasingly important to produce different parts of the automobile to reduce costs and weight, particularly in electrical vehicles [21]. In this context, the global composite materials market is expected to grow more than 4% during the period of 2022–2027 [22]. Therefore, given the importance of the composite sector in the polymer industry, the development of innovative materials and processes is crucial to fulfilling the requirements of novel applications [23].

Other important factors to consider in the preparation and selection of composite materials are their production cost and performance [24]. Therefore, any advancements in the technologies to prepare these materials will drive the growth of this market. Generally, the composites are prepared by means of a thermal curing either by autoclave process, resin transfer molding or by compression molding. However, these methods employ a lot of energy, due to the high temperatures and prolonged time of curing [25,26]. Therefore, to develop cost-effective and eco-friendly processes, it is necessary to prepare epoxy composites aimed to save energy.

Photopolymerizations are environmentally benign processes that exhibit advantages such as non-solvent reactions, low energy consumption, high polymerization rates, high productivity and lower reaction temperatures [27]. Due to the low penetration depth of the radiation used to induce these processes, they are commonly employed to produce thin films [28]. The photopolymerization is a versatile technique capable of initiating radical or cationic polymerization of vinyl monomers, acrylates, unsaturated esters, epoxy monomers and heterocycles, to name a few examples. In recent years this technique has been utilized to develop advanced applications such as 3D printing, photoresists and biomedical applications, among others [29,30,31,32,33].

The epoxy/thiol-ene photopolymerization (ETEP) is a rapid and efficient technique developed by our research group to cure epoxy resins [34,35]. In this method a thiol-ene system (TES) is added to an epoxy resin to initiate the curing process. The TES includes a curing agent and a multifunctional thiol—both in stoichiometric ratio—and a photoinitiator. The curing agent of ETEP is a compound with two or more allyl-functionalized tertiary amine groups in its structure, which can be of an aliphatic or a cycloaliphatic nature. The tertiary amine groups of the curing agent initiate the anionic ring opening polymerization of the epoxy resin, while the allyl groups in its structure can react with the thiol groups of the added multifunctional thiol, by means of the thiol-ene photopolymerization mechanism, producing polythioethers. As both types of polymerizations proceed simultaneously, a crosslinked chemically bonded polyether–polythioether co-network is obtained. The proposed mechanism for this technique has been discussed previously, elsewhere [34,36]. The ratio of polyethers–polythioethers can be tuned into the co-network adjusting the concentration of the TES in the photocurable formulation. In previous studies, the TES was varied from 20 mol% to 40 mol%, related to the moles of the epoxy resin, and an increase in the reactivity at higher concentrations was observed [34]. The obtained polythioethers are flexible species that improve the toughness of the co-network. The photopolymerization is considered an energy-saving process because it proceeds in a matter of a few minutes in comparison with hours, as in the case of the thermal curing of epoxy resins. The dual thermal-UV light curing conditions (85 °C-40 mW/cm^2^) of this technique have allowed us to prepare glass-fiber [37] as carbon-fiber epoxy [38] composite materials.

Thus, this study aims to produce epoxy bio-composites using a rapid and environmentally friendly process such as ETEP. A commercial biobased epoxy resin, a woven jute-cellulose fiber (WJF) fabric used as the reinforcement material, a biobased curing agent and a tetrafunctional thiol used as a crosslinker, were employed to produce the bio-composite material. The photocured bio-composites were analyzed by the three-point bending analysis, by dynamic mechanical analysis (DMA), scanning electron microscopy (SEM) and thermogravimetric analysis (TGA).

## 2. Experimental Methods

### 2.1. Materials

(3-mercaptopropyl) trimethoxysilane (MPTS); 2,2-dimethoxy-2-phenylacetophenone (DMPA); acetic acid; pentaerythritol tetrakis (3-mercaptopropionate) (PTKMP); hydrochloric acid and sodium hydroxide were purchased from Sigma-Aldrich, Toluca, Mexico, and used as received. The curing agent N^1^,N^1^,N^6^,N^6^-tetraallyl hexane-1,6-diamine (ALA4) was prepared as previously reported [34]. Information about synthesis and characterization of ALA4 is provided in Figure S1 in Supplementary Materials. The Greenpoxy 28 biobased epoxy resin was purchased from SICOMIN Inc. (Princeton, NJ, USA). The epoxy equivalent weight of this resin was 195–204. The woven jute fabric (WJF) was acquired from ULINE, Apodaca, NL, México.

### 2.2. Thiol-Functionalization of WJF

**Scheme 1:**
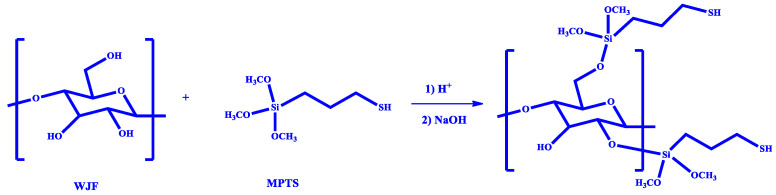
Silanization reaction of WJF with MPTS.

500 mL of distilled water were added to a beaker and heated to 50 °C. Once this temperature was reached, the pH of the water was adjusted to 4 with a 1.0 M HCl solution. Afterwards, 10 g of MPTS was added dropwise and the dispersion was vigorously stirred for 5 min. At the same time, a piece of WJF with dimensions of 60 × 40 cm was placed in a glass tray and subsequently the previously prepared dispersion of the silane was added. Next, the tray was placed in an ultrasonic bath for 2 h at 50 °C, flipping the fabric over after 1 h. After the ultrasound treatment, the pH was adjusted to 8 using a NaOH solution (0.5 M) and the radiation was kept for 2 h more. Upon the addition of the alkali solution, the resulting mixture displayed a white coloration that gradually vanished. Finally, the WJFs were washed with acetone to remove residual silane. A simplified representation of the involved chemical reaction is shown in Scheme 1

The degree of silanization was determined using the following equation:

Silanization% = [(W_1_ − W_0_)/W_0_] × 100 (1)

where W_0_ is the initial weight of the pristine WJF and W_1_ is the weight of modified WJF.

### 2.3. Photopolymerization of the Photocurable Formulation with the WJF

**Scheme 2:**
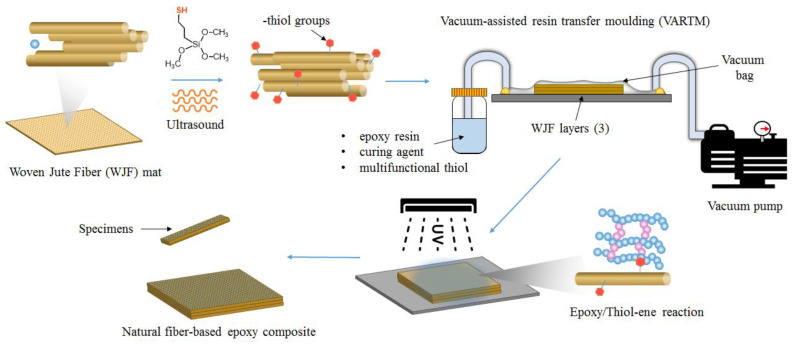
Method of preparation of bio-composites using the Epoxy/Thiol-ene Photopolymerization technique.

To produce the epoxy bio-composites a photocurable formulation was prepared by blending the biobased epoxy resin, the WJF and the TES, which was composed of the curing agent ALA4, PTKMP and the radical photoinitiator DMPA. The concentration of TES was varied from 30 mol% to 40 mol%. Table 1 illustrates the amounts of each component. The amounts of the components used for the formulations with non-functionalized (NF WJF) and thiol-functionalized fibers (SH WJF) were the same at the same concentration of TES. The biobased composites were prepared by the vacuum assisted resin transfer molding (VARTM) technique. The setup consisted of a base of a squared transparent plaque of tempered glass (40 cm × 40 cm and 1 cm thick). On top of this, two 30 cm × 13.5 cm sheets of infusion mesh, two pieces of 23 cm × 15 cm resin distribution media and then three layers of 20 cm × 12 cm fabric of WJF, were stacked on the tempered glass plaque. Four types of composites were prepared: two of them using 3 layers of untreated WJF (NF WJF) and the biobased epoxy resin with 30 mol% and 40 mol% of the TES; and two using three layers of thiol-functionalized WJF (SH WJF) with 30 mol% and 40 mol% of the TES. Before photocuring, the containers with the formulations were placed inside a vacuum chamber for 10 min to remove the air bubbles to minimize the void content in the composite. The photocurable formulation was then infused in the WJF fabric by the vacuum assisted resin transfer molding technique (VARTM). Thereafter, the assembly was introduced in an UV oven fitted with a 300 W UV Fusion lamp (Efsen UV & EB Technology, Holte, Denmark) for 30 min. The UV light intensity inside the oven was 40 mW/cm^2^, and the temperature was 85 °C due to the heat released by the UV lamp. Afterwards, the assembly was turned upside-down and irradiated another 30 min. After this period the sample was allowed to cool down and demolded. Scheme 2 depicts the methodology of this study to obtain the bio-composites.

### 2.4. Dynamic Mechanical Analysis (DMA) of the Composites

The obtained test specimens were analyzed using a Q800 dynamic mechanical analyzer TA Instruments (New Castle, DE, USA). The analysis was performed using the three-point bending clamp, in the range of 30 °C to 120 °C, using a frequency of 1 Hz and a heating rate of 5 °C/min. The glass transition temperature (T_g_) was considered as the maximum of the Tan *δ* curve.

### 2.5. Flexural Testing of WJF/Epoxy Composites

The three-point bending analysis was performed according to the ASTM D790 method. A united universal mechanical testing machine model SFM-1000 KN-E, with a load cell of 500 N, was employed. The tested specimens were loaded at 1.5 mm/min up to failure. For each formulation five samples were analyzed and the average is reported.

### 2.6. Scanning Electron Microscopy (SEM)

A JCM 6000 JEOL scanning electron microscope (Peabody, MA, USA) was used to analyze the interfacial adhesion between the jute fibers and the polymeric matrix. Samples were Au-Pd coated by chemical vapor deposition and then the analysis was performed under high vacuum condition at 15 kV.

### 2.7. X-ray Photoelectron Spectroscopy (XPS)

The XPS analysis was performed in a Thermo Scientific Escalab 250Xi X-ray Photoelectron Spectrometer (Sudbury, UK) using a 5 mg sample with a vacuum of 2 × 10^−8^ mTorr in the test chamber and a monochromatic X ray source with an aluminum anode and radiation energy of 1486.6 eV. Spectra were obtained with an energy pass of 117.4 eV and of 11.5 eV for the high-resolution spectra. The analysis region was 1400–0 eV of binding energy. In order to identify the chemical species on the surface of the fibers, C 1s were fitted using Gaussian functions. Prior to curve fitting, the spectra were baseline corrected using the Shirley function.

### 2.8. Thermogravimetric Analysis (TGA)

The thermal stability of the produced bio-composites was determined using a TA Instruments TGA Q500 (New Castle, DE, USA). Samples of 3–5 mg were accurately weighed and placed in the balance chamber of the equipment and analyzed at temperatures that ranged from 30 °C to 600 °C at a heating rate of 10 °C/min, in a high-purity nitrogen atmosphere at a flow rate of 50 mL/min

## 3. Results and Discussion

### 3.1. Characterization of Thiol Functionalized WJF

To improve the adhesion between the WJF and the polymeric matrix, it was necessary to introduce thiol groups on the surface of the jute fibers by means of a silanization reaction using MPTS. The thiol-functionalization of WJF enables its chemical bonding to the crosslinked polyether–polythioether crosslinked co-network produced during the ETEP. A modification of the straight-forward method of functionalization reported by Beaumont et.al. [39] was undertaken, where the silanization reaction proceeded under mild conditions. First, the akoxysilane MPTS was hydrolyzed with an acid aqueous solution to form the water-soluble silanol species. In the second step, the formed silanols condense with the hydroxyl groups in the cellulose fiber, using a basic medium, to form the Si–O–C bonds. Then, the functionalized WJF were washed with acetone to remove any non-covalently bounded silanes. The % of silanization achieved after drying the fibers to constant weight was 19%.

**Figure 1:**
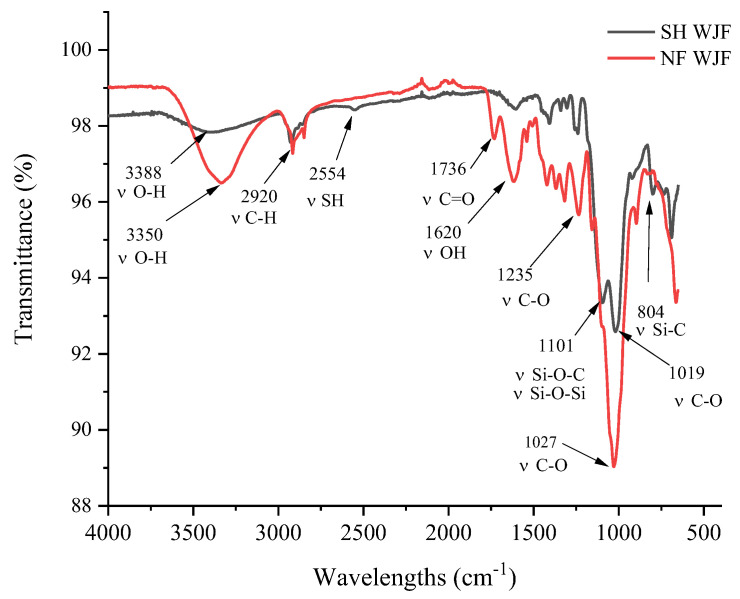
Comparison of the FTIR spectra of: NF WJF (red) and SH WJF (black). ν stands for stretching band.

The thiol-functionalized WJF were characterized by FTIR and XPS spectroscopies and by the EDS technique. Figure 1 depicts a comparison of the FTIR spectra of the NF WJF and the SH WJF. The untreated NF WJ shows the band at 3350 cm^−1^, characteristic of the stretching band of hydroxyl groups in the cellulose fibers. The weak band centered at 2920 cm^−1^ corresponds to the stretching band of C–H bonds. This band represents the proportion of the organic material content of the fiber. The small stretching band at 1735 cm^−1^ corresponds to the ester groups present in the pectine, that is a component of the natural plant fibers, while the band at 1626 cm^−1^ is characteristic of the adsorbed water in the cellulose fibers, along with the band at 1027 cm^−1^ which corresponds to the C–O bond. After functionalization with MPTS the resulting spectrum of SH WJF showed additional bands at 2554 cm^−1^ and 1101 cm^−1^, corresponding to the S–H bond and to the Si–O–C bond, respectively. These bands confirmed the thiol functionalization of WJF.

**Figure 2:**
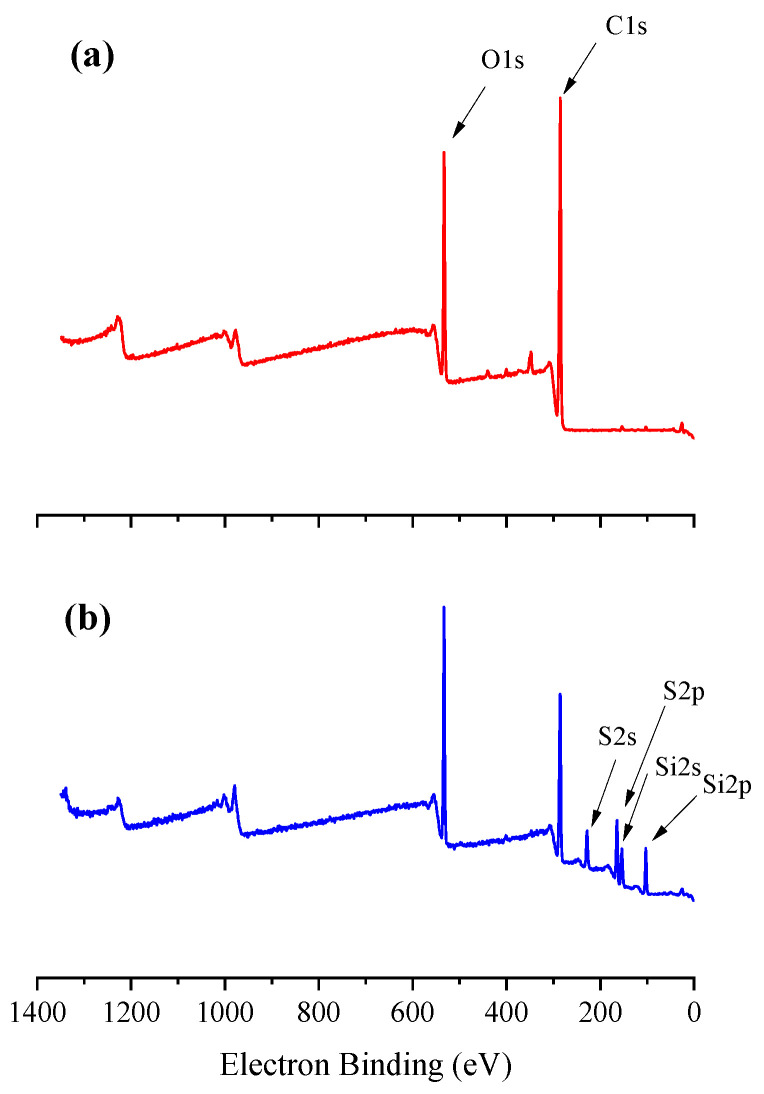
XPS survey of WJF before (**a**) and after MPTS treatment (**b**).

The XPS spectra of both untreated and thiol-functionalized WJF are shown in Figure 2. The XPS spectrum of untreated WJF shows two main peaks centered at 533 eV and at 286 eV, which corresponds to the O 1s and C 1s bonding, respectively. After the silanization treatment of WJF, the XPS spectrum of SH WJF (Figure 2b) shows new peaks appearing at 228 eV and 164 eV, which correspond to the S 2s and S 2p bonding. In addition, two new signals at 154 eV and 102 eV are attributed to the Si 2s and Si 2p bonding, respectively.

**Figure 3:**
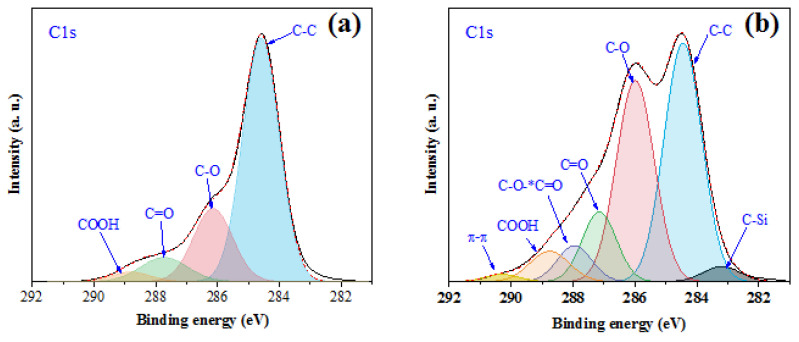
High resolution XPS spectra of the C 1s state for: (**a**) pristine WJF and (**b**) thiol-functionalized WJF. * denotes the carbon species that is responsible for that particular bonding energy.

The high resolution XPS spectra of the C 1s state of pristine WJF and of the thiol-functionalized WJF are depicted in Figure 3. The peak of C 1s of the pristine cellulose fibers is composed of several species as shown in Figure 3a. The maximum of the signal, centered at 284.5 eV correspond to the C–C bonding, followed by a shoulder at 286.2 eV characteristic of the C–O bonding. The second shoulder in the spectrum located at 287.7 eV corresponds to the C=O bonding while the shoulder at 288.9 eV can be ascribed to the COOH groups. These signals clearly indicate the organic nature of the natural fibers that are composed of cellulose, hemicellulose, lignin and pectin. Figure 3b depicts the XPS spectrum of WJF after the silanization reaction using MPTS. An increase in the intensity of the shoulder at 286.2 eV of the C–O bonding due to the transesterification reaction of the silanol groups of MPTS with the hydroxyl groups of cellulose was observed. It also detected the appearance of the C–Si bonding that confirms the successful silanization reaction.

The EDS semi-quantitative analysis of the fibers represents the third proof of the functionalization of the WJF with thiol groups. Table 2 shows a comparison of the elemental composition of untreated fibers and functionalized fibers (EDS spectra are shown in Figure S2). The elemental composition of SH WJF shows an increment in the mass% of Si and the appearance of S corresponding to the sulfur species S Ka and S Kb as well as of Si Ka, therefore confirming the silanization reaction to introduce thiol groups in the WJF. According to this analysis, the abundance of sulfur species in the functionalized fibers was of 3.2 wt%.

**Figure 4:**
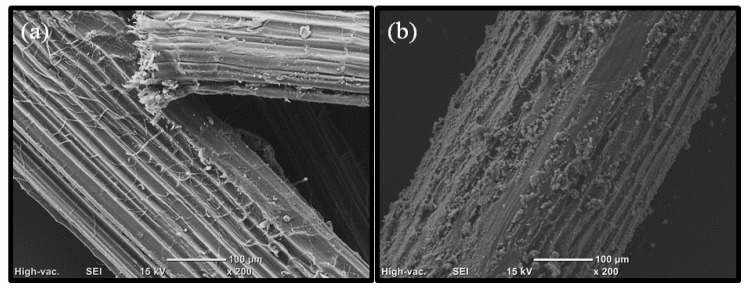
SEM micrographs of: (**a**) untreated WJF and (**b**) thiol-functionalized WJF.

SEM micrographs of the untreated WJF and the thiol functionalized WJF are depicted in Figure 4. The thiol-functionalized polysiloxane on the surface of the fibers resulting from the treatment with MPTS is shown in Figure 4b.

**Scheme 3:**
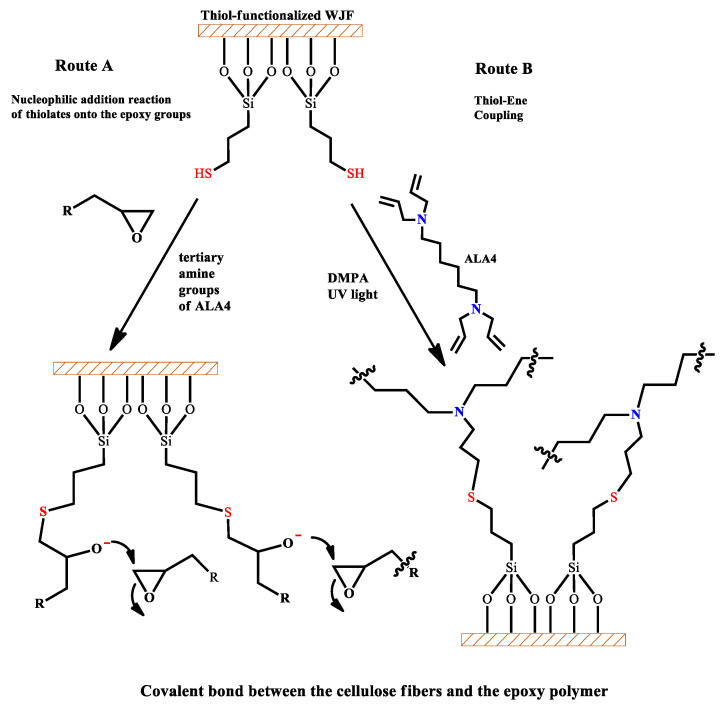
Reaction routes of the thiol groups introduced in the SH WJF to produce a covalent bonding with the polyether–polythioether polymeric matrix.

The thiol groups anchored to the WJF can interact with the components of the epoxy/thiol-ene photocurable formulation in two possible ways, as shown in Scheme 3. In Route A, the thiol groups in the fiber can react via an acid–base reaction with the highly basic tertiary amine groups of the curing agent ALA4, abstracting the proton of the thiol to produce thiolate groups. These nucleophilic species can attack the oxirane groups of the biobased epoxy resin to induce their anionic ring opening polymerization, producing polyethers. In Route B the thiol groups present in WJF can react with the allyl groups of ALA4 via the thiol-ene photopolymerization mechanism, to produce polythioethers. Thus, the thiol functionalization of WJF represents a convenient method to produce a covalent bonding between the cellulose fibers and the polymeric matrix.

### 3.2. Preparation of the Photocured Composites

The ETEP could be regarded as a very versatile technique that can be used to prepare fiber composite materials. This is not possible using a conventional radical or cationic photopolymerization due to the incomplete photopolymerization provoked by the low penetration of the light through the bulk of the assembly of the composite. In this study we were able to obtain the bio-composite by irradiating the assembly with UV light of 40 mW/cm^2^ at 85 °C. The 30 mol% and 40 mol% concentrations of TES were selected based on previous studies [34,35] in which we found that these formulations displayed the best ratio of reactivity/mechanical properties. At lower concentration of TES in the photocurable formulations, the rate of photopolymerization was too low, while at concentrations higher than 40%, the obtained material was very flexible due to the high proportion of polythioethers in the co-network. The obtained ratio of polymeric matrix to fiber was 52:48 in weight.

**Figure 5:**
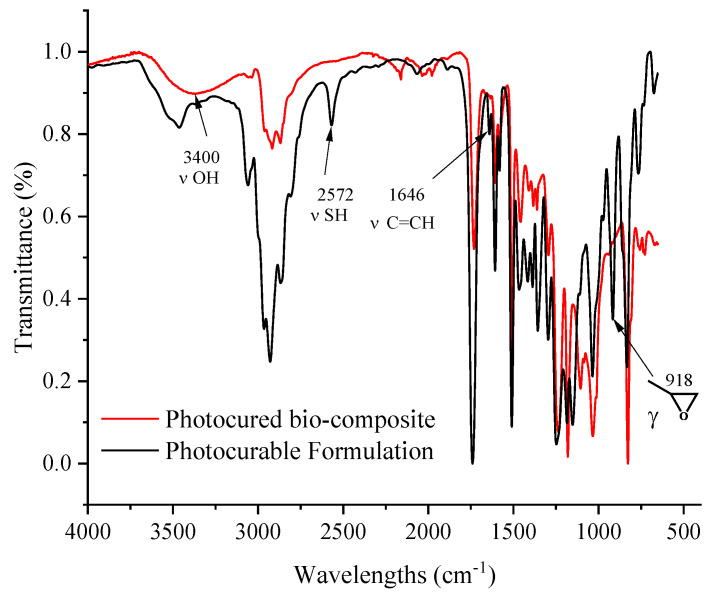
Comparison of the FTIR spectra of the photocurable formulation before UV irradiation and of the polymeric matrix of the bio-composite. ν and γ stands for stretching and bending bands.

**Scheme 4:**
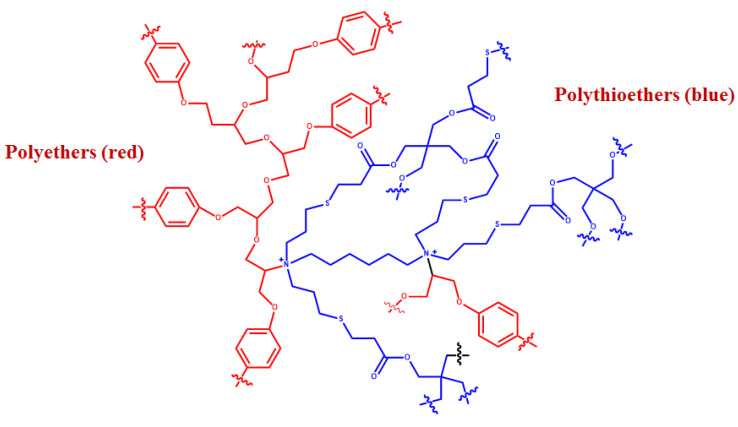
Simplified representation of the produced polyether–polythioether co-network.

Figure 5 depicts a comparison of the FTIR spectra of the photocurable formulation before being irradiated and of the polymeric matrix of the cured bio-composite. The decrease in the absorbance of the bands of the functional groups of the monomers involved in the ETEP, can be observed. The disappearance of the band at 918 cm^−1^, characteristic of the epoxy groups, together with the formation of a strong band at 3400 cm^−1^ that corresponds to the stretching band of the hydroxyl groups, indicates that the anionic ring opening polymerization of the epoxy groups to produce polyethers proceeded quantitatively. Additionally, the disappearance of the bands of the thiol group at 2572 cm^−1^ and of the band of the double bonds of ALA4 at 1646 cm^−1^ confirms the thiol-ene photopolymerization that results in the formation of polythioethers. Scheme 4 depicts a simplified representation of the polyether–polythioether co-network formed. Both networks are chemically bonded. The flexibility of the polythioethers imparts enhanced toughness to the co-network.

**Figure 6:**
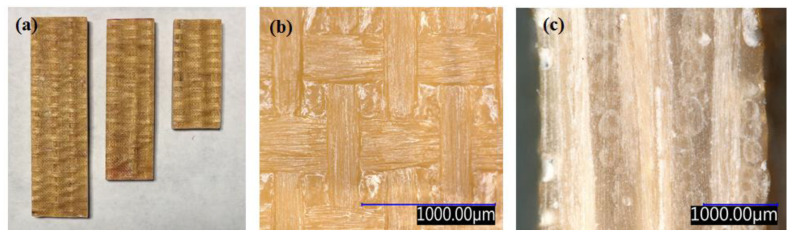
Photographs of the prepared test specimens using an optical microscopy: (**a**) photography of full-scale specimens, (**b**) micrograph at 200×, (**c**) transverse cut seen at 100×.

Figure 6a shows a photograph of the obtained test specimens used for the DMA and three-point bending analysis. Figure 6b shows a micrograph of the bio-composites that revealed a rather good distribution of the polymeric matrix in the WJF. A transverse cut of the bio-composite (Figure 6c) revealed that the fibers in the warp (0°) and weft (90°) directions, immersed in the polymeric matrix have a complete curing, an excellent wetting, good adhesion between the fiber and the polyether–polythioether co-network, as well as the absence of voids.

**Figure 7:**
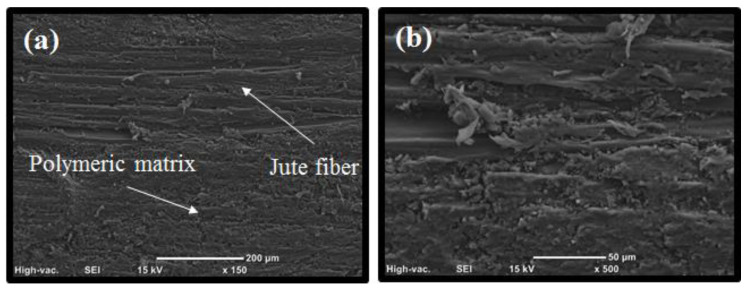
SEM micrographs of the transverse cut of the composite SH WJF40, (**a**): at 150 magnifications and (**b**) at 500 magnifications.

The latter was corroborated by means of SEM microscopy. Figure 7 shows micrographs of the photocured composite SH WJF40 at magnifications of 150× and 500×. As shown, the bio-composite exhibited a homogeneous dispersion along with excellent adhesion between the two phases of the material. A ductile fracture denoted by a leaf-like morphology was observed. This type of fracture is the result of the toughness induced by the presence of polythioethers in the crosslinked co-network.

### 3.3. Mechanical Properties of the WJF/Epoxy Composites

#### DMA Analysis

The viscoelastic and flexural properties of the obtained composites were examined by DMA and by the three-point bending test. Table 3 enlists a summary of the results of the DMA analysis and of the flexural properties of the materials (The obtained DMA curves are included as Figure S3 in Supplementary Materials). The results exhibited the high influence of the concentration of polythioethers in the crosslinked co-network of the obtained bio-composites, on the storage modulus and Tg values. Due to the lower concentration of the flexible polythioethers in the NF WJF30, the storage modulus was 3399 MPa against 2796 MPa of the NF WJF40, whereas the corresponding Tg values were of 76 °C and 70 °C, respectively. SH WJF30 displayed a storage modulus of 3356 MPa while for the SH WJF40 it was 2736 MPa, whereas the Tg were of 81 °C and 77 °C, respectively. Thus, the moduli of both untreated and treated WJF at the same concentration of polythioethers were very similar, indicating that the functionalization did not significantly impact those values while in the case of the Tg’s, the SH WJF30 and SH WJF40 displayed slightly higher values than their non-functionalized counterparts due to the chemical bond at the interface between the polymeric matrix and the cellulose fibers

**Figure 8:**
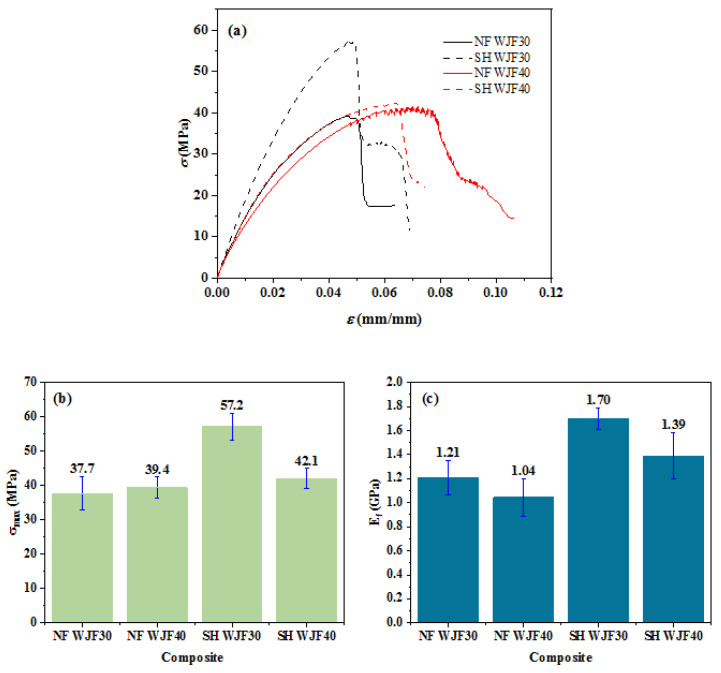
Results of the three-point bending analysis: (**a**) Representative curves *σ* vs. *ε* of the bio-composites, (**b**) *σ_max_* values for the composites, (**c**) *E_f_* values for the composites.

The flexural properties of the bio-composites are shown in Figure 8. Representative curves of load (*σ*) vs. displacement (*ε*) are depicted in Figure 8a. All composites exhibited a linear elastic behavior at low levels of deformation, then a change in the slope identified the plastic deformation of the composites, followed by failure. It is also important to mention that none of the composites displayed an abrupt failure, characteristic of brittle materials. Instead, the composites slowly failed, starting with microcracks in the matrix, followed by the propagation of the failure either in the matrix or at the fiber/matrix interface. In the cases of the samples NF WJF40 and SH WJF40, this was particularly noticeable as a zig-zag trend at the end of the curves ascribed to a stepwise crack propagation instead of a sudden failure. The flexible polythioethers in the co-network are the responsible species of the toughening mechanism of the composites. As seen in Figure 8a, NF WJF40 and SH WJF40 displayed higher levels of strain due to the higher content of polythioethers compared with NF WJF30 and SH WJF30. Figure 8b shows that the average flexural strength (*σ_max_*) of NF WJF 30 and NF WJF40 were 37.7 MPa and 39.4 MPa, respectively. The results of flexural strength are similar to those reported in other studies of epoxy bio-composites [40,41]. The bio-composites SH WJF30 and SH WJF40 displayed values of flexural strength of 57.2 MPa and 42.1 MPa, respectively. Figure 8c shows the comparison of the flexural modulus (*E_f_*) of the composites. It was found that the functionalized composites SH WJF30 and SH WJF40 displayed higher values of *E_f_* (1.70 GPa and 1.39 GPa, respectively) in comparison with the non-functionalized composites.

By analyzing the results shown in Figure 8b,c it was found that the SH WJF30 composite increased ~52% and ~40% in *σ_max_* and *E_f_* with respect to NF WJF30, while the SH WJF40 composite showed an increase of ~7.0% in *σ_max_* and of ~34% in *E_f_* over NF WJF40. These improvements found in the composites with silane-functionalized fibers were attributed to the enhanced fiber/matrix interface. The composite SH WJF30 displayed higher values of flexural strength and flexural modulus than SH WJF40 because the former is more rigid due to the lower concentration of polythioethers in the co-network, therefore a greater amount of energy is required to bend the test specimens. Given the above results, it was concluded that the composite SH WJF30 displayed the best combination of flexural and viscoelastic properties, resulting in a material with good mechanical properties combined with excellent toughness.

**Figure 9:**
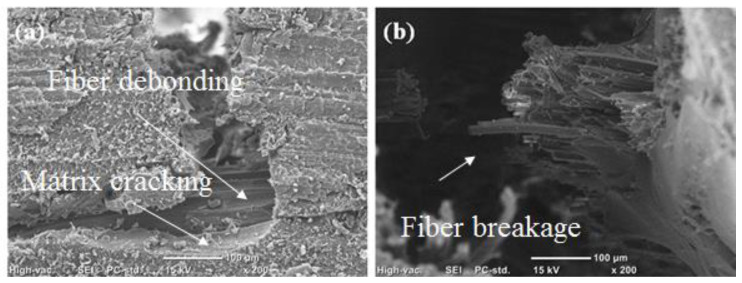
Comparison of SEM micrographs of: (**a**) NF WJF30 and (**b**) SH WJF30.

In order to get insight of the governing failure mechanism of the composites, the SEM analysis was carried out on fracture surfaces of NF WJF30 and SH WJF30 and the results are shown in Figure 9.

The NF WJF30 composite failed due to the matrix cracking followed by fiber debonding (Figure 9a) while the composite SH WJF30 failed due to fiber breakage (Figure 9b). Fiber debonding in NF WJF30 is a characteristic signature of a poor fiber/matrix interface. Since the matrix has a weak interaction with fiber, there is not an efficient load transfer yielding first matrix cracking followed by fiber debonding. When there is a good fiber/matrix bonding, the load is efficiently transferred from the matrix to the fiber, as shown in the micrograph of Figure 7b of the composite SH WJF30, where fiber breakage seems to be the main failure mechanism. Those results are in good agreement with the mechanical analysis, in which the composites containing functionalized fibers exhibited the highest *σ_max_* and *E_f,_* due to an enhanced fiber/matrix interface.

### 3.4. Thermal Stability of the Bio-Composites

**Figure 10:**
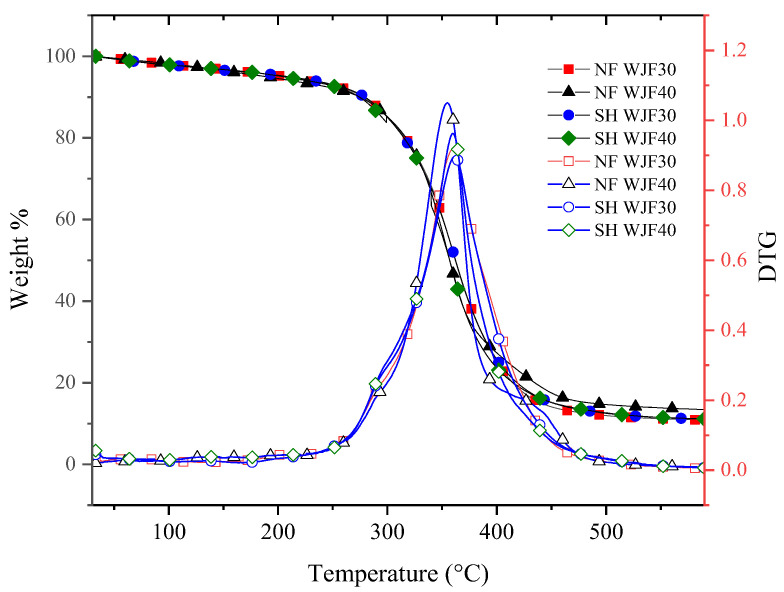
Results of the TGA analysis of the obtained bio-composite.

The obtained composites were analyzed by TGA to determine their thermal stability. As shown in Figure 10, all the composites displayed similar behavior when heated in nitrogen atmosphere, which could be attributed to the similar nature of the polymeric matrix, differing only in the concentration of polythioethers in the crosslinked co-network. This type of behavior was previously reported, where polyether–polythioethers co-networks at different concentrations of polythioethers displayed similar thermal degradation curves [42]. The weight loss in the range 100 °C–105 °C was of 2.7%, which corresponds to the content of humidity in the composite. The onset of the thermal degradation—considered as the temperature at which there is a weight loss of 5%—was of 205 °C and 200 °C for NF WJF30 and NF WJF40 and of 210 °C and 204 °C for SH WJF30 and SH WJF40, respectively. The DTG curves in Figure 7b also shows similar results with maximum rates of degradation in the range 354 °C–358 °C. These experiments indicate that the obtained bio-composites display good thermal stability.

## 4. Conclusions

A novel kind of bio-composite with a polyether–polythioether matrix and WJF reinforcement was developed by using a photopolymerization process. With the aim to chemically bond the fibers to the polymeric matrix, the WJF were functionalized with thiol groups using MPTS, allowing a better interfacial adhesion. In this regard, the thiol-functionalized WJF composites displayed better flexural properties than the composite based on non-functionalized fibers. The latter was attributed to the chemical bonding between the cellulose fibers with the polymeric matrix. The influence of the content of polythioethers in the crosslinked co-network was evidenced by the higher modulus and T_g_ observed of SH WJF30 than its counterpart SH WJF40, due to the lower concentration of the flexible polythioethers. Considering all the obtained data, it was concluded that the composite SH WJF30 displayed the best combination of mechanical properties. These kinds of epoxy bio-composites can be useful in eco-friendly structural applications.

## Figures and Tables

**Table 1:** Components of the epoxy/thiol-ene photocurable formulations.

Sample SH WJF 30	Equivalents	mmol	grams
Greenpoxy 28	1	250.0	86.0
ALA4	0.3	76.0	37.0
PTKMP	0.3	76.0	20.9
DMPA	0.002	1.5	0.38
**Sample SH WJF 40**	**Equivalents**	**mmol**	**grams**
Greenpoxy 28	1	220.0	75.0
ALA4	0.4	88.0	43.1
PTKMP	0.4	88.0	24.3
DMPA	0.002	2.0	0.45

The formulations with non-functionalized fibers (NF WJF X) used the same amounts that formulations SH WJF X.

**Table 2:** Comparison of the results of the EDS semi-quantitative analysis of: (a) NF WJF and (b) SH WJF.

Element	NF WJF	SH WJF
	Mass%	Mass%
C	58.86	51.64
O	12.76	12.49
Al	0.67	1.78
Si	0.53	2.39
S	-------	3.20
Ca	2.39	1.12
Pd	10.44	11.08
Au	14.35	16.3
Total	100%	100%

**Table 3:** Summary of viscoelastic and flexural properties of the obtained bio-composites.

Sample	Storage Modulus (MPa)	Tg (°C)	Flexural Strength (*σ_max_*) MPa	Flexural Modulus (*E_f_*) GPa
NF WJF30	3399	76	37.7	1.21
NF WJF 40	2796	70	39.4	1.04
SH WJF30	3356	81	57.2	1.70
SH WJF40	2736	77	42.1	1.39

## Supplementary Materials

The following supporting information can be downloaded at: https://www.mdpi.com/article/10.3390/polym15010060/s1, Figure S1: Synthesis and characterization of N1,N1,N6,N6-tetraallyl hexane-1,6-diamine (ALA4), Figure S2: EDS Results; Figure S3: DMA results of the obtained bio-composites: (a) Storage Modulus vs. Temperature curves, (b) Tan δ vs. Temperature curves.
