# Supplementary material for: Fabrication of Woven Jute Fiber Epoxy Bio-Composites through the Epoxy/Thiol-Ene Photopolymerization Technique
Source: Polymers (Basel). 2022 Dec 23;15(1):60. doi: 10.3390/polym15010060 (PMC9824046; doi:10.3390/polym15010060)
Supplement: Supplementary file 1 [file polymers-15-00060-s001.zip › polymers-2089946-supplementary.pdf]

Supplementary Information

Fabrication of Woven Jute Fiber Epoxy Bio-Composites through the Epoxy/Thiol-Ene Photopolymerization Technique

Ricardo Acosta Ortiz*, Roberto Yañez Macías, José de Jesús Ku Herrera and Aida Esmeralda García Valdez

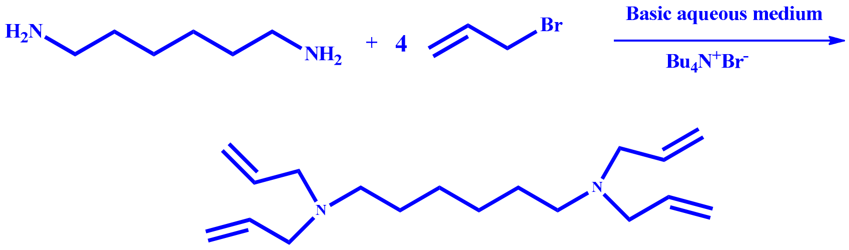

(**a**) The method to prepare the ALA4 is described in reference 34.

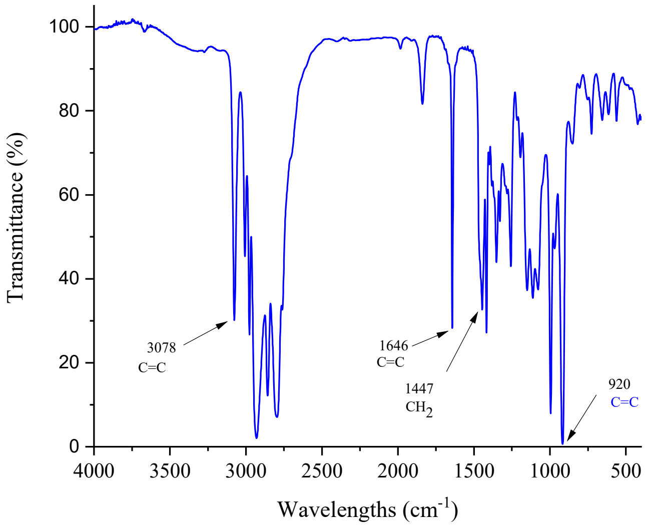

(**b**) FTIR spectrum of ALA4.

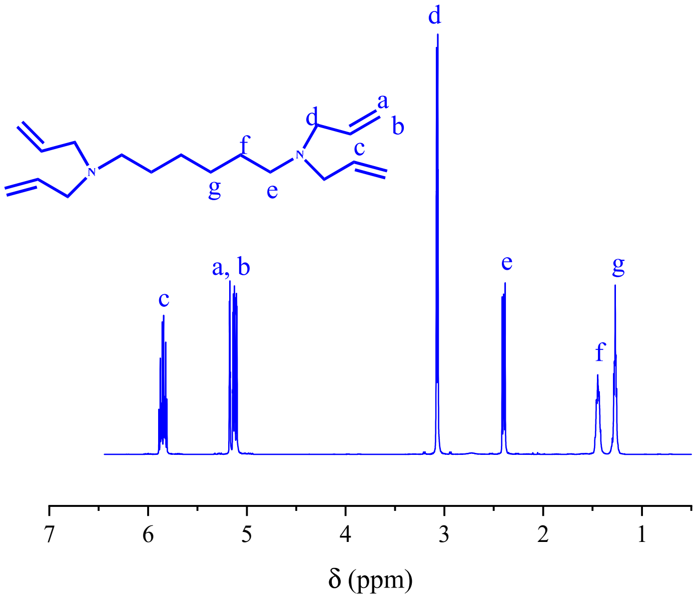

(**c**) ^{1}H NMR Spectrum of ALA4.

Figure S1. Synthesis and characterization of N1, N1, N6, N6-tetraallyl hexane-1,6-diamine (ALA4).

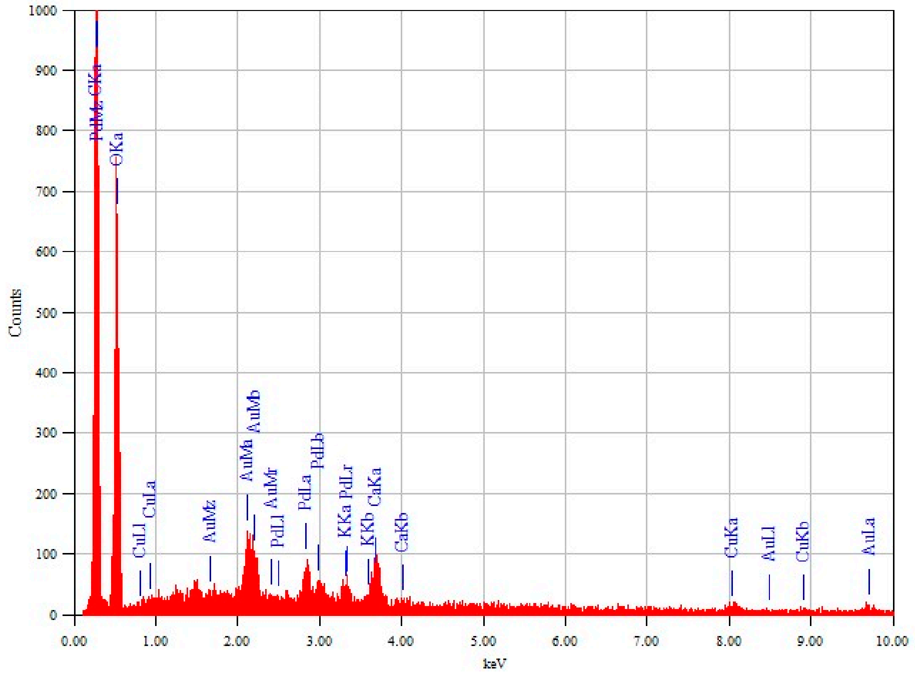

(**a**) EDS spectrum of the NF WJF

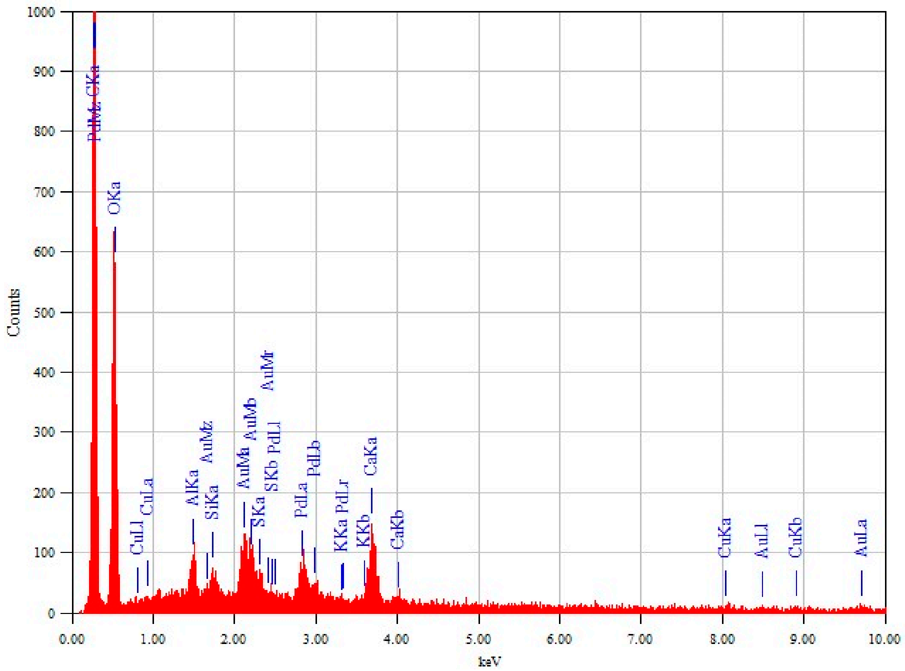

(**b**) EDS spectrum of the SH WJF.

Figure S2. EDS Results.

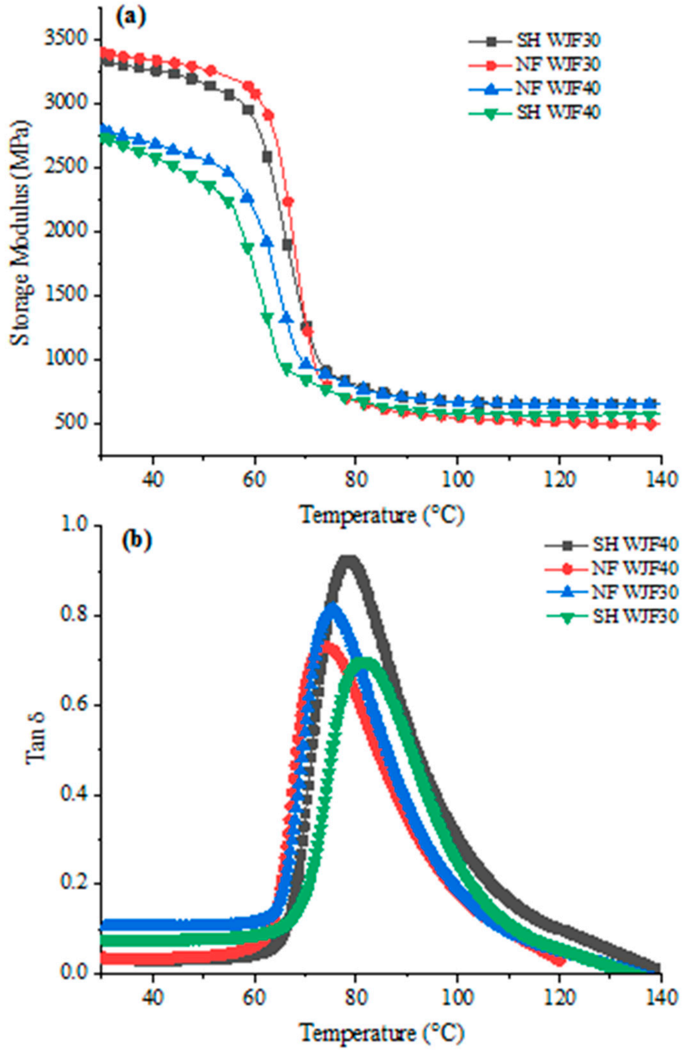

Figure S3. DMA results of the obtained bio-composites: (**a**) Storage Modulus vs. temperature curve; (**b**) Tan δ vs. Temperature curves.
